# GammaTile® (GT) as a brachytherapy platform for rapidly growing brain metastasis

**DOI:** 10.1093/noajnl/vdad062

**Published:** 2023-05-30

**Authors:** Rajiv Dharnipragada, Clara Ferreira, Rena Shah, Margaret Reynolds, Kathryn Dusenbery, Clark C Chen

**Affiliations:** University of Minnesota Medical School, University of Minnesota Twin-Cities, Minneapolis, Minnesota, USA; Department of Radiation Oncology, University of Minnesota, Minneapolis, Minnesota, USA; Department of Oncology, North Memorial Health, Robbinsdale, Minnesota, USA; Department of Radiation Oncology, University of Minnesota, Minneapolis, Minnesota, USA; Department of Radiation Oncology, University of Minnesota, Minneapolis, Minnesota, USA; Department of Neurosurgery, University of Minnesota, Minneapolis, Minnesota, USA

**Keywords:** brachytherapy, brain metastasis, GammaTile, glioblastoma, rapid regrowth

## Abstract

**Background:**

A subset of brain metastasis (BM) shows rapid recurrence post-initial resection or aggressive tumor growth between interval scans. Here we provide a pilot experience in the treatment of these BM with GammaTile® (GT), a collagen tile-embedded Cesium 131 (^131^Cs) brachytherapy platform.

**Methods:**

We identified ten consecutive patients (2019–2023) with BM that showed either (1) symptomatic recurrence while awaiting post-resection radiosurgery or (2) enlarged by >25% of tumor volume on serial imaging and underwent surgical resection followed by GT placement. Procedural complication, 30-day readmission, local control, and overall survival were assessed.

**Results:**

For this cohort of ten BM patients, 3 patients suffered tumor progression while awaiting radiosurgery and 7 showed >25% tumor growth prior to surgery and GT placement. There were no procedural complications or 30-day mortality. All patients were discharged home, with a median hospital stay of 2 days (range: 1–9 days). 4/10 patients experienced symptomatic improvement while the remaining patients showed stable neurologic conditions. With a median follow-up of 186 days (6.2 months, range: 69–452 days), no local recurrence was detected. The median overall survival (mOS) for the newly diagnosed BM was 265 days from the time of GT placement. No patients suffered from adverse radiation effects.

**Conclusion:**

Our pilot experience suggests that GT offers favorable local control and safety profile in patients suffering from brain metastases that exhibit aggressive growth patterns and support the future investigation of this treatment paradigm.

**Key Points**

1. From this cohort of ten brain metastases (BM) patients, GammaTile (GT) had no procedural complications, no 30-day mortality, and a median overall survival of 265 days.
2. GT offers favorable local control and safety profile in patients suffering from BM.

**Importance of the Study**

GammaTile® (GT) is a brachytherapy platform consisting of Cesium-131 seeds embedded with an absorbable collagen matrix that received US FDA clearance to treat recurrent brain tumors in late 2018. Here, we report our experience with ten consecutive patients (2019–2023) with rapidly growing brain metastases. There were no procedural complications or 30-day mortality. All patients were discharged home, with a median hospital stay of 2 days (range: 1–9 days). With a median follow-up of 186 days (6.2 months), no local recurrence was detected. The median overall survival (mOS) for this patient cohort was 265 days. There were no adverse radiation effects in this series. This pilot experience suggests that GT offers favorable local control (LC) and safety profile in patients suffering from brain metastases that exhibit aggressive local growth patterns and supports future investigation of this treatment paradigm.

Brain metastasis (BM) afflicts up to 30% of all cancer patients and is a major cause of cancer morbidity.^1–3^The available literature suggests that these tumors are rapidly proliferating, averaging 1%–2% volumetric growth per day.^4–6^ In addition, there are subsets of BM that exhibit a particularly aggressive pattern, with a growth rate that is an order of magnitude higher than the average.^7^ The molecular nature of these subsets of BM remains poorly characterized, though there is evidence suggesting that these tumors are enriched for cancer stem cells which are particularly radiation resistant.^8,9^ Moreover, these rapidly growing tumors are more likely to cause neurologic decline, requiring surgical interventions that potentially delay or interrupt life-saving systemic therapy.^10^ Achieving LC in these rapidly growing BM is, therefore, of clinical importance.

GammaTile® (GT, GT Medical Technologies Inc., Tempe AZ) is a Food and Drug Administration (FDA) cleared form of brachytherapy where 4 gamma-emitting ^131^Cs seeds (half-life of 9.7 days) are embedded within a 2 × 2 cm bioresorbable collagen sponge (Figure 1A and B). GT is designed for application to the resection cavity at the time of the tumor resection. The collagen sponge is sufficiently supple to conform to the morphology of the resection cavity but rigid enough to avoid the collapse of the resection cavity (Figure 1C). The former property allows conformal radiation delivery while the latter minimizes morbidity risk associated with overlapping radiation fields, potentially increasing the risk for radiation necrosis. The ^131^Cs seeds deliver 120–150 Gy at the contact point with rapid dose fall-off thereafter—yielding 60–80 Gy at 5 mm depth. We previously demonstrated the safety of this brachytherapy platform in recurrent glioblastoma patients.^11–13^

**Figure 1.**
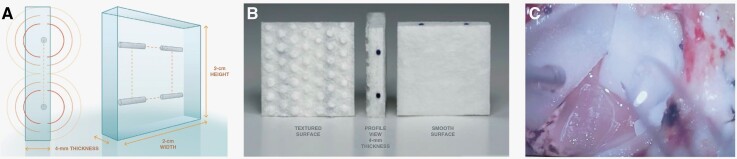
GammaTile®. **A** Schematic dimensions of collagen matrix and cesium seeds, **B** shown are the cesium seeds embedded in the collagen matrix with asymmetric positioning for correct seed-brain surface distance, and **C** Tiles after hydration and placement into the resection cavity.

Here, we examine the safety profile and the capacity of GT to achieve LC for a subset of BM that showed either (1) symptomatic regrowth after initial gross total resection or (2) showed >25% growth on serial MRIs. These patients underwent surgical resection and GT placement. LC in this setting is of particular interest since the aggressive nature of these tumors should maximally challenge the therapeutic efficacy of brachytherapy.

## Methods

### Study Population

The study was conducted under IRB 00010486 (University of Minnesota Human Research Protection Program). Data were retrospectively collected from patients who underwent first-time or repeat surgery following initial resection due to significant tumor regrowth. Consecutive cases between 2019 and 2023 were identified. All cases were reviewed in a multi-disciplinary brain tumor board (neurosurgery, neuroradiology, radiation oncology, neuro-oncology, and neuro-pathology). During the consent for the GT implant, the radiation oncologist and medical physicist were present and recommended precautions for radiation safety. Data collected included: patient age, sex, diagnosis, symptoms at presentation of recurrence, time from last surgery, tumor type, tumor location, length of hospital stay, postoperative neurologic deficits, duration of follow-up, wound complications, 30-day readmission, and survival (measured from the time of GT placement). Volumetric determinations were done using Medtronic Stealth.

### GT Brachytherapy and Neurosurgical Techniques

The GT units required were estimated in the following manner: the tumor volume was contoured on the preoperative T1 MRI with gadolinium, and the resection cavity surface area (cm^2^) was calculated after excluding the skull-facing surface. This surface area was divided by 4 cm^2^ (GT cross-sectional area), yielding an estimate for the required GT. Room survey, GT transfer, and GT placement were as previously described.^14^ In brief, a medical physicist transported the packages of GT units in radiation-shielded trays. A scrubbed radiation oncologist retrieved GTs from the packages, placed them in sterile metallic trays, and hydrated the units. These hydrated GTs were placed into the resection cavity by the neurosurgeon. A radiation safety survey was conducted before and after the completion of the procedure.

For determination of the post-implant GT dosimetry, a 1 mm, thin-cut CT, and T1 MRI with gadolinium of the brain were obtained 24–48 hours post-procedure. The following parameters were determined: High-risk clinical target volume (HR-CTV, defined as the regions including 5 mm expansion of the resection cavity), V_50_ HR-CTV (volume receiving 30 Gy), V_100_ HR-CTV (volume receiving 60 Gy), and V_150_ HR-CTV (volume receiving 90 Gy), and D90 HR-CTV (dose delivered to 90% of the HR-CTV, see Supplementary Table 1).^11,15^ LC was defined as the absence of new T1 contrast enhancement within HR-CTV.

Surgical techniques were as previously described.^12^ All GT-implants were performed by the senior author (CCC). Discharge disposition is based on physical therapy and occupational therapy recommendation. A postoperative visit with the surgeon takes place 2–3 weeks after the surgery. The patient is seen by the radiation oncologist and medical/neuro-oncologist approximately 1 month after the surgery and 2–3 months thereafter. There was no loss in follow-up.

### Statistical Analysis

The mean, median, and range of surgical data and mOS of the cohort are calculated using *R software*. Overall survival is plotted using Kaplan–Meier plots.

## Results

### Definition of Rapidly Growing BM

In this study, we defined rapid growth as (1) symptomatic recurrence while awaiting post-resection radiosurgery or (2) enlarged by >25% of the initial tumor volume on serial imaging. While BM growth while awaiting radiosurgery is expected, symptomatic recurrence while awaiting radiosurgery is rare. Our departmental policy is to complete postoperative SRS of the BM resection bed by 4 weeks post-surgery. Our record review indicates <5% of my BM patients who underwent resection suffer symptomatic regrowth during this 4-week period. For these patients with symptomatic recurrence, the tumor growth during the postoperative period reaches >25% of the original tumor volume.

Customary to the clinical practice of the senior author, a brain MRI is performed immediately prior to surgery for the purpose of stereotaxis. Patients whose BM grew by >25% when comparing this preoperative MRI with the initial presenting MRI were selected for analysis in this study (patients 5–10).

### Patient Characteristics

The demographics of the ten BM patients who underwent surgical resection followed by GT implant are shown in Table 1. Two patients in this cohort (patients 1 and 2) suffered symptomatic recurrence within 4 weeks of resection while waiting for radiosurgery to the resection cavity. Patient 3 suffered a *Staphylococcus aureus* wound infection from the initial surgery. After completing 6 weeks of antibiotics, the patient presented to the emergency room with recurrent right hemiparesis. MRI revealed significant regrowth of the left frontal BM. Since surgical resection is warranted given the patient’s recurrent symptoms and radiation to the resection cavity has yet to be administered, the option of surgery with GT implant and surgery followed by postoperative radiosurgery were discussed with the patient. The patient opted for the former option. One patient (patient 4) suffered tumor recurrence after previous resection and radiosurgery, with >25% volumetric tumor growth with MRI’s 1 month apart. The remaining 5 patients presented with newly diagnosed BM with >25% volumetric tumor growth with MRIs 1 month apart. The cohort is male predominant (7 males and 3 females). The histologic distribution is comparable to other published series, with a predominance of lung cancer.^16,17^ The cohort’s mean age was 64.6 (range: 53–79). One patient (patient 7) underwent an awake craniotomy while the remaining patient population underwent craniotomy under general anesthesia.

**Table 1.** Patient characteristics

Patient	Age	Sex	Indications^*^	Diagnosis	Tumor location	Postoperative condition	Hospital stay (days)	30-day readmission	Local Failure	Follow-up (days)	Survival after GT placement (days)	Cause of death
1	71	M	1	Squamous cell carcinoma	Right frontal	Resolved hemiparesis	5	None	No	119	119	Systemic disease progression
2	58	M	1	Renal cell carcinoma	Right temporal	Stable	2	None	No	265	265	Systemic disease progression
3	59	M	1	Small cell lung cancer	Left frontal	Improved hemiparesis	4	Wound infection	No	79	79	Systemic disease progression
4	74	M	2	Non-small cell lung cancer	Right frontal	Stable	1	None	No	452	452	Alive
5	73	F	3	Melanoma	left temporal	Stable	9	Urinary tract infection	No	69	69	Systemic disease progression
6	57	M	3	EML4-ALK non-small cell lung cancer	Right parietal	Improved left-hand function	2	None	No	339	339	Alive
7	59	F	3	Breast cancer	Right frontal	Improved left manual dexterity	2	None	No	235	235	Alive
8	79	F	3	Non-small cell lung cancer	Right frontal	Stable	3	None	No	137	137	Alive
9	53	M	3	Non-small cell lung cancer	Right temporal	Stable	2	None	No	280	280	Alive
10	63	M	3	Melanoma	left parietal	Stable	1	None	No	137	137	Alive

^*^Indications: 1 = BM recurrence while awaiting radiosurgery, 2 = BM recurrence after resection and radiosurgery and showed >25% growth on serial MRI, 3 = newly diagnosed BM with >25% growth on serial MRI.

### Extent of Resection and GT Dosimetry

As shown in Supplementary Table 1, the residual enhancing region in the T1 pre-gadolinium and post-gadolinium were identical for 9/10 patients, suggesting gross total resection. Patient 7 underwent an awake craniotomy, and a tumor tightly adherent to a cortical vein was intentionally left to minimize the risk of neurologic injury. The average resection cavity volume was 12.28 cm^3^ (range: 0.4–26.8). The median number of GTs implanted was 6 (range: 1–8). High-risk clinical target volume (HR-CTV) in all cases was defined as regions including 5 mm expansion of the resection cavity and received 60 Gy. The average D_90_ HR-CTV (dose received by 90% of the HR-CTV) was 51.2 Gy (range: 22.4–80.8 Gy). The average V_50_ HR-CTV (volume receiving 50% of the prescribed 60 Gy (or 30 Gy)), V_100_ HR-CTV (volume receiving 100% of the prescribed 60 Gy), and V_150_ HR-CTV (volume receiving 150% of the 60 Gy (or 90 Gy)) were: 15.8 cm^3^ (range: 3.6–28.1), 13.4 cm^3^ (range: 0.9–24.2), and 7.9 cm^3^ (range: 0.2–16.5), respectively.

### Illustrative Example 1: Rapidly Recurring BM after Surgical Resection

**Figure 2.**
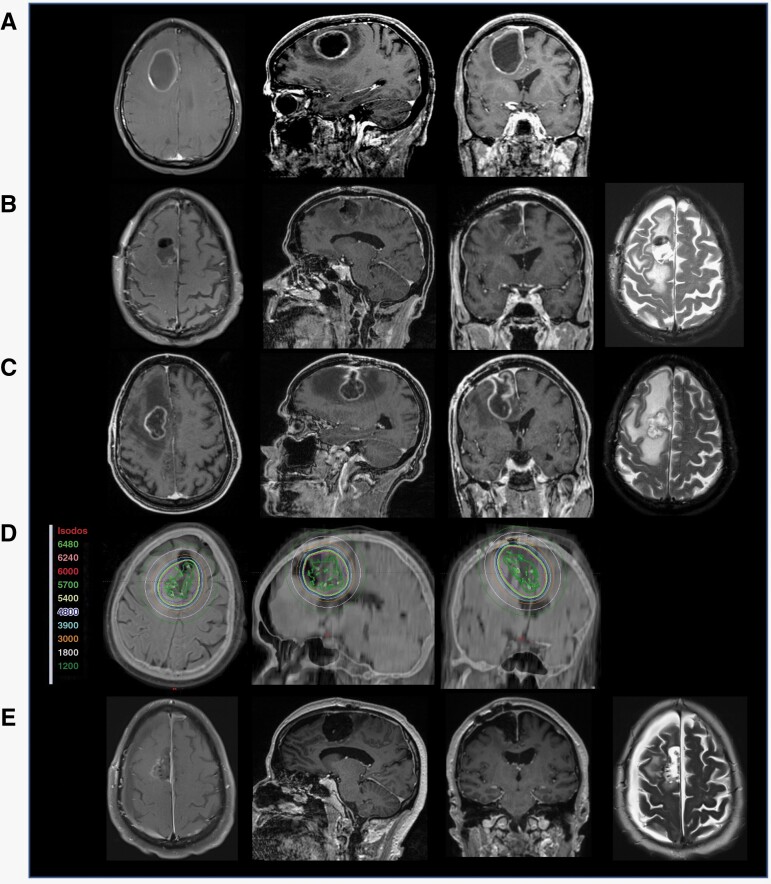
Illustrative example: rapidly recurring BM after surgical resection. **A** MRI with a right frontal contrast-enhancing lesion in a patient with stage IV squamous cell carcinoma, **B** The patient presented 22 days post-resection with recurrent left hemiparesis. MRI shows regrowth of contrast-enhancing lesion before the patient could undergo radiosurgery, **C** postoperative MRI following re-resection, and **D** Post-op T1 MRI and CT image registration displaying the GammaTile placement in the resection cavities with isodose lines (color-coded numbers represent isodoses in centigray = cGy), **E** 3-months follow-up MRI without evidence of local recurrence.

Patient 1 is a 71-year-old male with stage IV squamous cell cancer who presented with progressive left hemiplegia. MRI of the brain showed a right frontal 4.5 × 3.6 × 4.5 cm peripherally enhancing mass with extensive peri-lesional FLAIR abnormality (Figure 2A). The patient underwent gross total resection of the lesion. Intraoperative pathology revealed findings consistent with metastatic squamous cell carcinoma. The patient was discharged home with a plan for resection cavity radiosurgery but returned on postoperative day 22 with recurrent left hemiparesis. MRI of the brain showed regrowth of the contrast-enhancing lesion (measuring 3.7 × 2.5 × 2.8 cm, Figure 2B). Without complication, the patient underwent a repeat gross resection (Figure 2C) and GT placement (Figure 2D). The left hemiparesis resolved post-operatively, and the patient was discharged home on postoperative day 5. There is no evidence of local recurrence at the 3-month follow-up MRI (Figure 2E). However, the patient subsequently succumbed to systemic disease progression.

### Illustrative Example 2: Rapidly Growing Recurrent BM Post-radiosurgery

**Figure 3.**
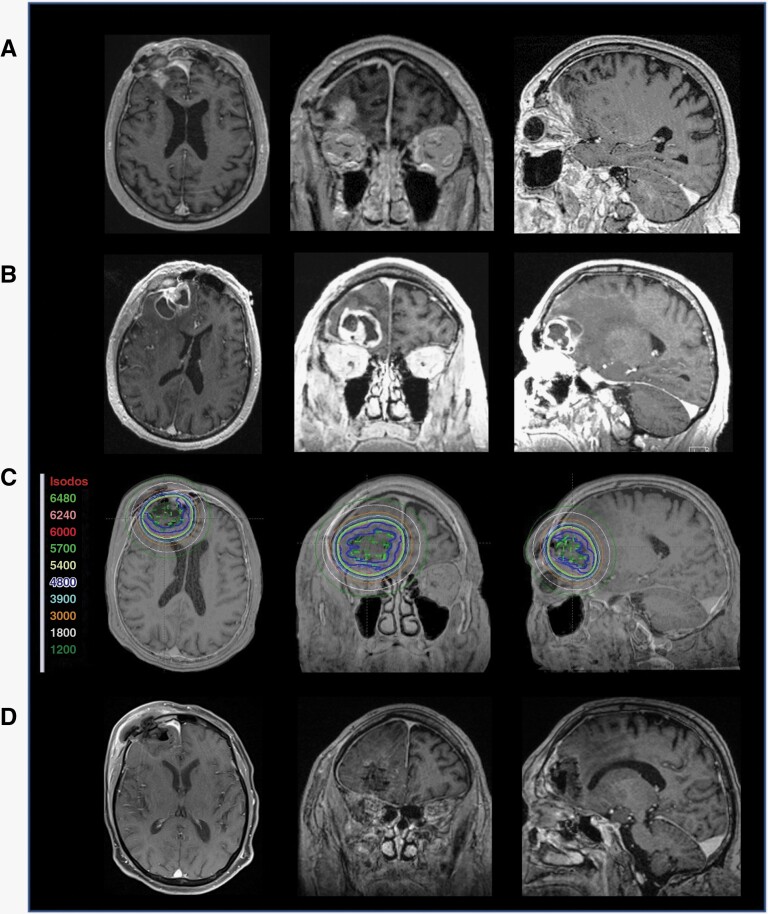
Illustrative example: rapidly growing recurrent BM post-radiosurgery. **A** Surveillance MRI of a patient with stage IV NSCLC taken 8 months after resection of a right frontal mass followed by radiosurgery to the resection cavity. **B** Rapid enlargement of the right frontal lesion approximately 1 month after the initial MRI. **C** Post-op T1 MRI with contrast and CT image registration displaying the GammaTile placement in the resection cavities after resection of both lesions with isodose lines (color-coded numbers represent isodoses in centigray = cGy). **D** 14 months follow-up MRI post-GT placement showing no evidence of local recurrence or adverse radiation effect.

Patient 4 is a 74-year-old male with stage IV non-small cell lung cancer (NSCLC) status post gross total resection of right frontal BM and radiosurgery to the resection cavity. Eight months after the radiosurgery, a new contrast enhancement measuring 1.1 × 1.9 × 2.3 cm with minimal surrounding FLAIR signals (Figure 3A). MRI taken a month after this MRI showed significant enlargement of the contrast-enhancing region, now measuring 2.9 × 3.4 × 2.8 cm and significant peri-lesional FLAIR signals (Figure 3B). The patient underwent gross total resection of the lesion, with pathology revealing findings consistent with metastatic carcinoma. GT was placed at the time of surgery (Figure 3C), and the patient was discharged home on postoperative day 2. There is no evidence of local recurrence at the 14-month follow-up MRI (Figure 3D).

### Illustrative Example 3: Radiation Treatment of BM Without Significant Interruption of Targeted Therapy

**Figure 4.**
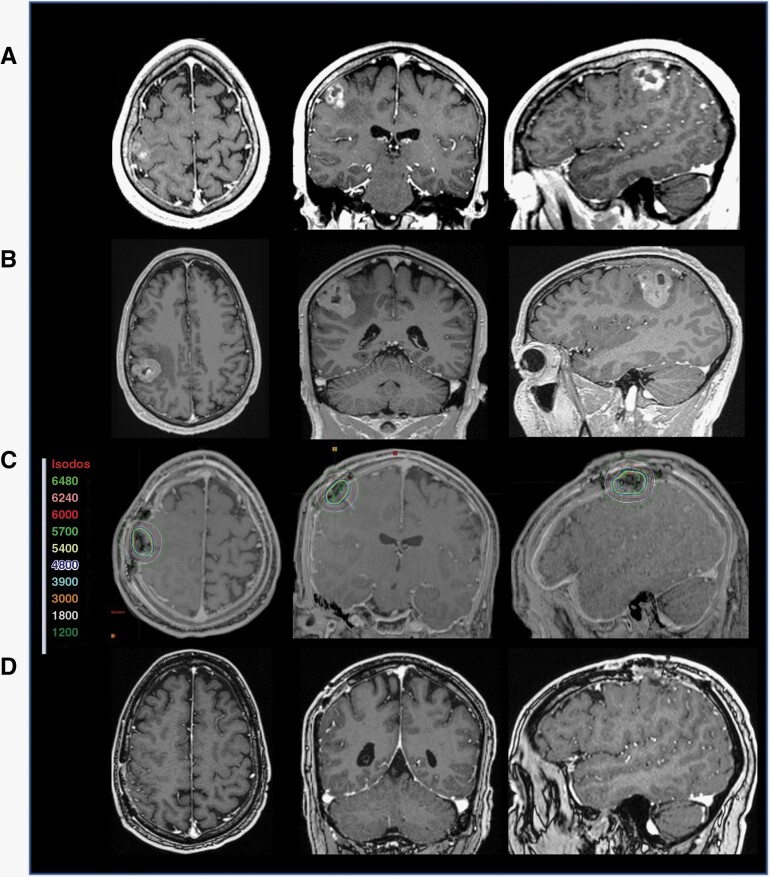
Illustrative example: radiation treatment of BM without significant interruption of targeted therapy. **A** MRI of a patient with EML4-ALK rearranged NSCLC who presented with progressive right-hand function. **B** MRI performed on the morning of surgery 2 weeks after initial MRI showing >25% increase in tumor volume. **C** Post-op T1 MRI with contrast and CT image registration displaying the GammaTile placement in the resection cavities after resection of both lesions with isodose lines (color-coded numbers represent isodoses in centigray = cGy). **D** Six months follow-up MRI post-GT placement showing no evidence of local recurrence or adverse radiation effect. The patient received alectinib, an ALK-targetingi therapy, up until a day prior to surgery and resumed therapy a day after the procedure without complications.

Patient 6 is a 57-year-old male with stage EML4-ALK rearranged NSCLC who presented with progressive right-hand function. Serial MRI showed a right parietal contrast-enhancing lesion measuring 2.0 × 2.0 × 2.5 cm on the initial MRI and 3.6 × 3.1 × 3.4 cm taken on the day of surgery (2 weeks after the initial MRI, Figure 4A and B). The patient underwent gross total resection of the lesion, with intraoperative pathology revealing findings consistent with metastatic carcinoma. GT was placed at the time of surgery (Figure 4C). The patient experienced an improved left-hand function and was discharged home on postoperative day 2. Of note, the patient received alectinib, a molecular therapy targeting ALK, up until a day prior to surgery and resumed therapy a day after the procedure without complications. There is no evidence of local recurrence at the 1-year follow-up MRI (Figure 4D).

### Illustrative Example 4: Subtotal Resection of a Rapidly Growing BM

**Figure 5.**
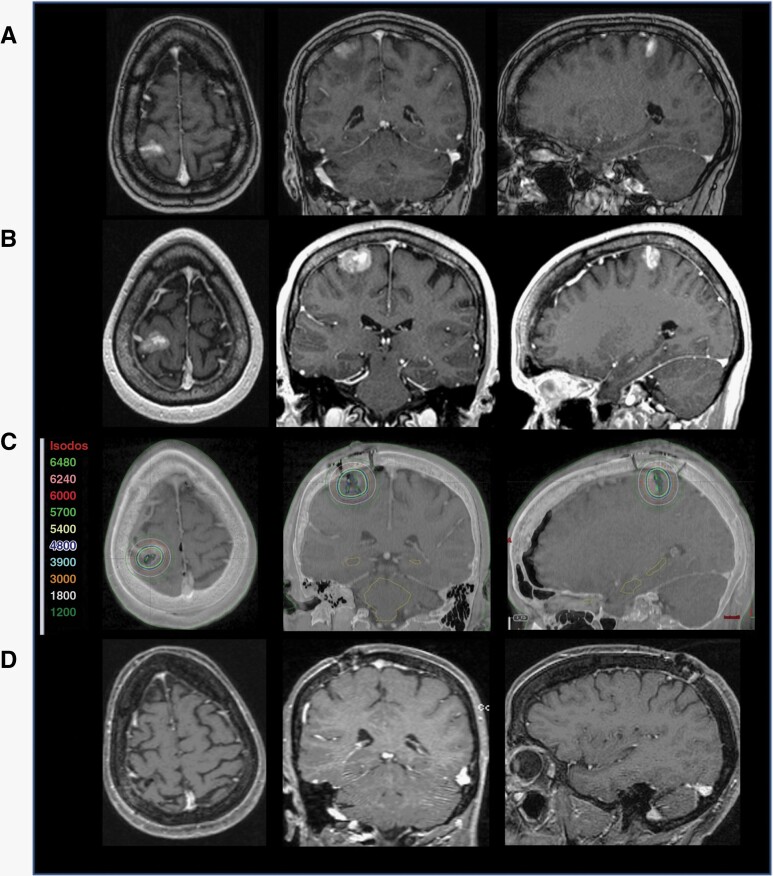
Illustrative example: subtotal resection of a rapidly growing BM. **A** MRI of a stage IV breast cancer patient who presented with progressive worsening of left-hand dexterity. **B** MRI performed on the morning of surgery 2 weeks after initial MRI showing >25% increase in tumor volume. **C** Post-op T1 MRI with contrast and CT image registration displaying the GammaTile placement in the resection cavities after resection of both lesions with isodose lines (color-coded numbers represent isodoses in centigray = cGy). **D** Twelve months follow-up MRI post-GT placement showing no evidence of local recurrence or adverse radiation effect. The patient received alectinib, an ALK-targetingi therapy, up until a day prior to surgery and resumed therapy a day after the procedure without complications.

Patient 7 is a 59-year-old female with stage IV breast cancer who presented with progressive worsening of left-hand dexterity. Serial MRI showed a contrast-enhancing lesion with epicenter in the right motor strip measuring 0.9 × 1.8 × 1.2 cm on the initial MRI and 2.4 × 1.8 × 1.9 cm on the MRI taken 1 month afterward (Figure 5A and B), with an associated complaint of worsening left manual dexterity. The patient underwent an awake craniotomy with the goal of gross total resection. Intraoperative pathology revealed findings consistent with metastatic carcinoma. Due to the adherence of the tumor to a sizable, en passage cortical vein, a small amount of residual tumor was intentionally left on the venous surface. GT was placed immediately adjacent to the residual tumor (Figure 5C). The patient reported improvement in her left-hand dexterity and was discharged home on postoperative day 2. There is no evidence of contrast enhancement at the 3- or 6-month follow-up MRI (Figure 5D).

### Hospital Course, Readmission, and LC

Four patients (4/10; 40%; patients 1, 3, 6, and 7) experienced neurologic improvement post-surgical resection/GT implant. All other patients remained neurologically unchanged post-resection/GT placement. There was no procedural complication. All patients were discharged home with a median hospital stay of 2 days (range: 1–9 days). There was no 30-day mortality. There were two 30-day readmissions: 1 urinary tract infection (8.3%, patient 5) and 1 wound infection (8.3%, patient 3). Of note, the patient with the infected wound had suffered a previous wound infection before the GT implantation, and the pathogenic organism for both infections was the same (*S. aureus*).

With a median follow-up of 186 days (6.2 months, range: 69–452 days), no local recurrence was detected. The mOS for this patient cohort was 265 days from the time of GT placement (Supplementary Figure 1). There was no MR evidence (including expansion of FLAIR or contrast enhancement) in or adjacent to the HR-CTV that would suggest acute radiation effects in any of the treated patients during follow-up.

## Discussion

In this study, we share our pilot experience of GT as a radiation delivery platform for BM that exhibits locally aggressive growth patterns. For the 3 patients who underwent repeat resection and GT placement within a month of the original surgery, the safety profile in this experience is of particular interest. This pilot experience suggests a favorable safety profile in this context, with 1 single infection in a patient who is likely colonized with pathogenic *S. aureus* from a prior infection. Consistent with prior studies, GT implantation did not appear to prolong hospitalization, significantly increase postoperative morbidity, or elevate the risk of 30-day readmission.^12^ The absence of local recurrence given the median follow-up is notable given the aggressive nature of the brain tumor treated. The favorable overall survival is equally notable in this context.

The illustrative examples serve to demonstrate the potential clinical benefits of GT. Illustrative example 1 demonstrates that symptomatic recurrence occurs in a subset of BM patients between the time of resection and radiosurgery. While the majority of post-resection radiosurgery takes place within a month of the procedure, up to a third of the BM undergoes radiosurgery >2 months post-surgery, with some patients never receiving the standard of care resection cavity radiosurgery.^18^ GT implant at the time of surgery ensures timely radiation to the resection cavity. Illustration example 2 suggests that GT may be an effective salvage therapy for tumors that recur after previous radiosurgery, an observation supported by a recent case series.^19^ Illustrative example 3 provides proof of the principle that molecularly targeted therapy can be administered with minimal interruption in combination with a GT implant. Illustrative example 4 suggests the efficacy of GT against macroscopic, residual BM. Finally, the wound complication of patient 3 raises caution in GT use in patients with previous wound infections. Of note, reasonable overall survival was achieved despite the aggressive growth of the BM. Given the limited case series here, however, validations of these considerations are warranted in future studies.

As a pilot series, the study validity benefits from the non-exclusion of patients through a consecutive patient series. Since GT was approved only in 2018 and as a single institutional experience of a subset of highly aggressive BM, the sample size is necessarily limited. This limited sample size and the heterogeneity of malignant tumor histology limit the interpretation of the results reported here. The study nevertheless focuses on a patient population whose BM exhibited an unusually aggressive local growth pattern. To our knowledge, our study represents the first to explore the outcome of GT as a radiation delivery platform in this context. The safety and efficacy profile of GT observed in our pilot study appears favorable and warrants future prospective and randomized studies in terms of validation.

## Conclusion

Here we report a consecutive case series documenting the outcome of GT implant as a radiation delivery platform in patients suffering from BMs with aggressive local growth patterns. Our safety and LC data are favorable and support the future investigation of GT in this unique clinical context.

## Supplementary Material

vdad062_suppl_Supplementary_Figure_S1

vdad062_suppl_Supplementary_Table_S1

## References

[1] Nayak L , Lee EQ, Wen PY. Epidemiology of brain metastases. Curr Oncol Rep. 2012; 14(1):48–54. 22012633 10.1007/s11912-011-0203-y

[2] Cagney DN , Martin AM, Catalano PJ, et al. Incidence and prognosis of patients with brain metastases at diagnosis of systemic malignancy: a population-based study. Neuro Oncol. 2017; 19(11):1511–1521. 28444227 10.1093/neuonc/nox077 PMC5737512

[3] Wen PY , Loeffler JS. Management of brain metastases. Oncology (Williston Park). 1999; 13(7):941–957. 10442342

[4] Kobets AJ , Backus R, Fluss R, Lee A, Lasala PA. Evaluating the natural growth rate of metastatic cancer to the brain. Surg Neurol Int. 2020; 11:254. 33024592 10.25259/SNI_291_2020 PMC7533080

[5] Yoo H , Jung E, Nam BH, et al. Growth rate of newly developed metastatic brain tumors after thoracotomy in patients with non-small cell lung cancer. Lung Cancer. 2011; 71(2):205–208. 20570390 10.1016/j.lungcan.2010.05.013

[6] Yoo H , Nam BH, Yang HS, et al. Growth rates of metastatic brain tumors in nonsmall cell lung cancer. Cancer. 2008; 113(5):1043–1047. 18618515 10.1002/cncr.23676

[7] Niwińska A , Murawska M, Pogoda K. Breast cancer subtypes and response to systemic treatment after whole-brain radiotherapy in patients with brain metastases. Cancer. 2010; 116(18):4238–4247. 20549816 10.1002/cncr.25391

[8] Hintelmann K , Petersen C, Borgmann K. Radiotherapeutic strategies to overcome resistance of breast cancer brain metastases by considering immunogenic aspects of cancer stem cells. Cancers (Basel). 2022; 15(1):211. 36612206 10.3390/cancers15010211 PMC9818478

[9] Debeb BG , Xu W, Woodward WA. Radiation resistance of breast cancer stem cells: understanding the clinical framework. J Mammary Gland Biol Neoplasia. 2009; 14(1):11–17. 19252973 10.1007/s10911-009-9114-z

[10] Liu Q , Tong X, Wang J. Management of brain metastases: history and the present. Chin Neurosurg J. 2019; 5:1. 32922901 10.1186/s41016-018-0149-0 PMC7398203

[11] Brachman DG , Youssef E, Dardis CJ, et al. Resection and permanent intracranial brachytherapy using modular, biocompatible cesium-131 implants: results in 20 recurrent, previously irradiated meningiomas. J Neurosurg. 2019; 131(6):1819–1828. 10.3171/2018.7.JNS18656 30579269

[12] Gessler DJ , Neil EC, Shah R, et al. GammaTile® brachytherapy in the treatment of recurrent glioblastomas. Neurooncol Adv. 2021; 4(1):vdab185. 35088050 10.1093/noajnl/vdab185 PMC8788013

[13] Gessler DJ , Ferreira C, Dusenbery K, Chen CC. GammaTile®: surgically targeted radiation therapy for glioblastomas. Future Oncol. 2020; 16(30):2445–2455. 32618209 10.2217/fon-2020-0558

[14] Ferreira C , Sterling D, Reynolds M, et al. First clinical implementation of GammaTile permanent brain implants after FDA clearance. Brachytherapy. 2021; 20(3):673–685. 33487560 10.1016/j.brachy.2020.12.005

[15] Chen AM , Chang S, Pouliot J, et al. Phase I trial of gross total resection, permanent Iodine-125 brachytherapy, and hyperfractionated radiotherapy for newly diagnosed glioblastoma multiforme. Int J Radiat Oncol Biology Phys. 2007; 69(3):825–830. 10.1016/j.ijrobp.2007.03.061 17512132

[16] Pham A , Yondorf MZ, Parashar B, et al. Neurocognitive function and quality of life in patients with newly diagnosed brain metastasis after treatment with intra-operative cesium-131 brachytherapy: a prospective trial. J Neurooncol. 2016; 127(1):63–71. 26650067 10.1007/s11060-015-2009-5

[17] Wernicke AG , Smith AW, Taube S, et al. Cesium-131 brachytherapy for recurrent brain metastases: durable salvage treatment for previously irradiated metastatic disease. J Neurosurg. 2017; 126(4):1212–1219. 27257835 10.3171/2016.3.JNS152836

[18] Roth O’Brien DA , Kaye SM, Poppas PJ, et al. Time to administration of stereotactic radiosurgery to the cavity after surgery for brain metastases: a real-world analysis. J Neurosurg. 2021;135(6):1695–1705. 10.3171/2020.10.JNS201934 34049277

[19] Imber BS , Young RJ, Beal K, et al. Salvage resection plus cesium-131 brachytherapy durably controls post-SRS recurrent brain metastases. J Neurooncol. 2022; 159(3):609–618. 35896906 10.1007/s11060-022-04101-9 PMC9328626

