# Supplementary figures and images for: GammaTile® (GT) as a brachytherapy platform for rapidly growing brain metastasis
Source: Neurooncol Adv. 2023 May 30;5(1):vdad062. doi: 10.1093/noajnl/vdad062 (PMC10263112; doi:10.1093/noajnl/vdad062)

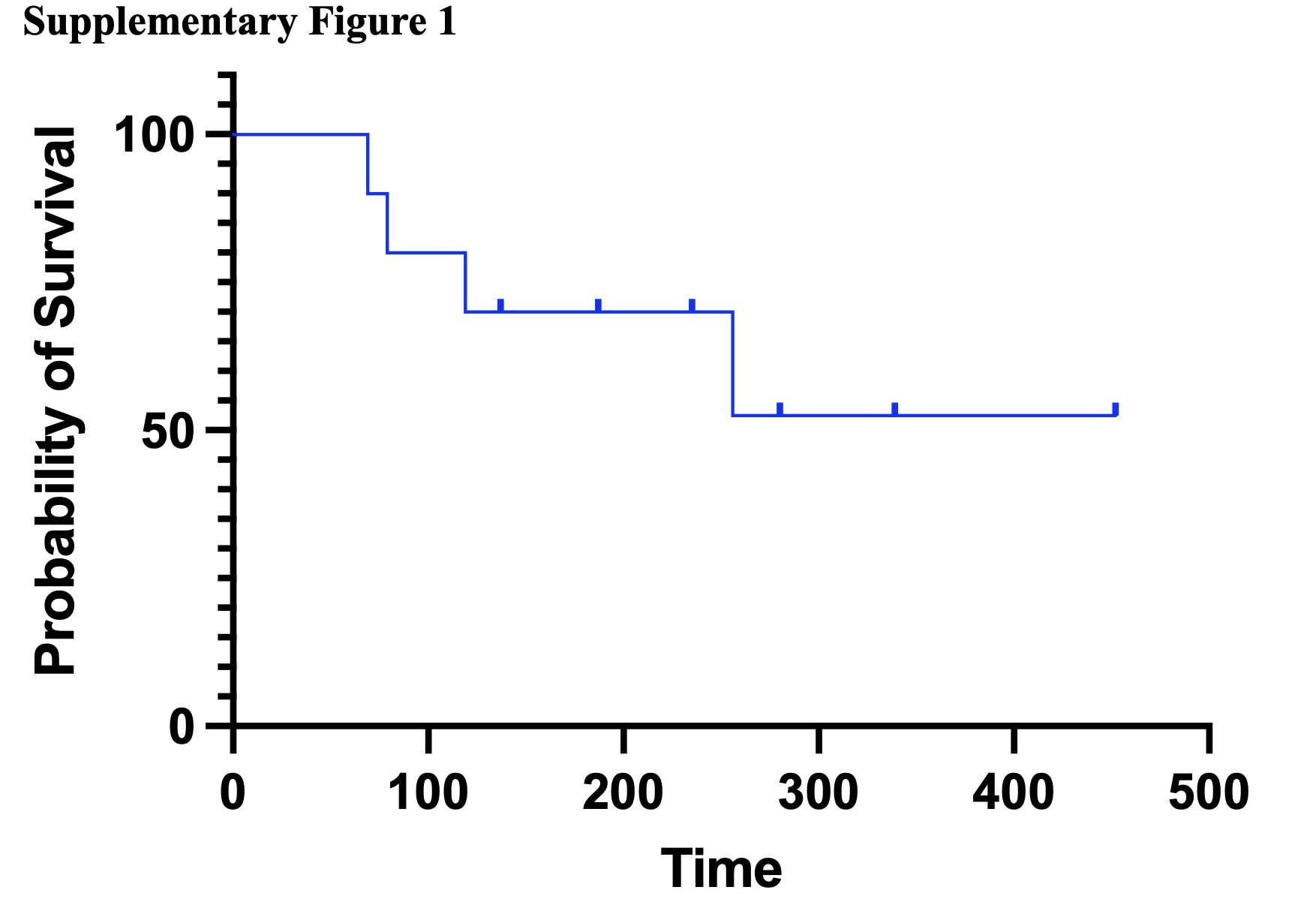

Supplement: vdad062_suppl_Supplementary_Figure_S1 [file vdad062_suppl_supplementary_figure_s1.jpeg]
