# Supplementary material for: GammaTile® (GT) as a brachytherapy platform for rapidly growing brain metastasis
Source: Neurooncol Adv. 2023 May 30;5(1):vdad062. doi: 10.1093/noajnl/vdad062 (PMC10263112; doi:10.1093/noajnl/vdad062)
Supplement: vdad062_suppl_Supplementary_Table_S1 [file vdad062_suppl_supplementary_table_s1.docx]

| **Supplementary Table 1.**  **GammaTile Dosimetry Parameters** | | | | | | | | | | | | |
| --- | --- | --- | --- | --- | --- | --- | --- | --- | --- | --- | --- | --- |
| Patient | Lesions | Resection cavity volume (cm^3^) | Number of Tiles Implanted | GTVr  enhancement on MRI T1 without gadolinium (cm^3^) | GTVr  enhancement on MRI T1 with  gadolinium (cm^3^) | PTV (HRCTV) (cm^3^) | Prescription Dose  (Gy) | Prescription depth into brain parenchyma (mm) | D90 PTV (HRCTV) (Gy) | V50 PTV  (30 Gy) (HRCTV)  (cm^3^) | V100 PTV  (60 Gy) (HRCTV)  (cm^3^) | V150 PTV  (90Gy) (HRCTV) (cm^3^) |
| 1 | 1 | **15.8** | **6** | **1.1** | **1.1** | **28.1** | **60** | **5** | **57** | **28.1** | **24.17** | **11.13** |
| 2 | 1 | **15.5** | **6** | **2.6** | **2.6** | **18** | **60** | **5** | **59.85** | **18** | **16.18** | **9.41** |
| 3 | 1 | **18.5** | **6** | **0.3** | **0.3** | **21.2** | **60** | **5** | **63.9** | **21.2** | **19.78** | **10.99** |
| 4 | 1 | **21.5** | **6** | **0** | **0** | **18.3** | **60** | **5** | **47.56** | **18.3** | **13.22** | **6.12** |
| 5 | 1 | **12.1** | **7** | **0** | **0** | **20** | **60** | **5** | **80.82** | **20** | **19.66** | **16.51** |
| 6 | 1 | **2.1** | **1** | **0** | **0** | **5.2** | **60** | **5** | **22.35** | **3.58** | **0.87** | **0.24** |
| 7* | 1 | **0.4** | **1** | **0** | **1.2** | **4.7** | **60** | **5** | **36.91** | **4.46** | **2.97** | **1.57** |
| 8 | 1 | **2.6** | **2** | **0.4** | **0.4** | **8.6** | **60** | **5** | **26.91** | **7.54** | **2.83** | **0.91** |
| 9 | 1 | **26.8** | **8** | **0** | **0** | **23.3** | **60** | **5** | **71.33** | **23.3** | **22.54** | **15.55** |
| 10 | 2 | **7.5** | **3.5** | **0** | **0** | **14** | **60** | **5** | **45.41** | **13.69** | **11.44** | **7.56** |
| *Sub-total resection  Residual gross tumor volume (GTVr), Planned target volume (PTV) High-risk clinical target volume (HRCTV), Dose received by 90% of the volume (D90), Volume receiving 50% (30Gy) of the prescribed dose (V50), Volume receiving 100% (60Gy) of the prescribed dose, Volume receiving 150% (90Gy) of the prescribed dose | | | | | | | | | | | | |
